# *In Vitro* Antibacterial Activity of Pomegranate Juice and Peel Extracts on Cariogenic Bacteria

**DOI:** 10.1155/2017/2152749

**Published:** 2017-10-25

**Authors:** Gianmaria Fabrizio Ferrazzano, Elisa Scioscia, Daniela Sateriale, Gabiria Pastore, Roberta Colicchio, Chiara Pagliuca, Tiziana Cantile, Brunella Alcidi, Marco Coda, Aniello Ingenito, Elena Scaglione, Annunziata Gaetana Cicatiello, Maria Grazia Volpe, Michele Di Stasio, Paola Salvatore, Caterina Pagliarulo

**Affiliations:** ^1^Department of Neuroscience, Reproductive and Oral Sciences, Section of Paediatric Dentistry, University of Naples Federico II, Via S. Pansini, No. 5, 80131 Naples, Italy; ^2^Department of Science and Technology, Sannio University, Via Port'arsa, No. 11, 82100 Benevento, Italy; ^3^Department of Molecular Medicine and Medical Biotechnology, Federico II University Medical School, Via S. Pansini, No. 5, 80131 Naples, Italy; ^4^CEINGE, Advanced Biotechnologies s.c.ar.l., Via Gaetano Salvatore, No. 486, 80145 Naples, Italy; ^5^Institute of Food Science-CNR, Via Roma, No. 64, 83100 Avellino, Italy

## Abstract

**Aim:**

To evaluate the antimicrobial activity of hydroalcoholic extracts of pomegranate (*Punica granatum *L.) peel and juice, against the microorganisms considered the main etiologic agents of dental caries.

**Methods:**

The values of the minimum inhibitory concentration (MIC) and the minimum bactericidal concentration (MBC) were determined against* Streptococcus mutans *Clarke ATCC® 25175™ strain and* Rothia dentocariosa *clinical isolate.

**Results:**

Peel extracts inhibit effectively the growth and survival of* S. mutans *ATCC 25175 strain and* R. dentocariosa *clinical isolate with MIC and MBC values of 10 *μ*g/*μ*l and 15 *μ*g/*μ*l, respectively. Furthermore, the pomegranate juice extract showed high inhibitory activity against* S. mutans *ATCC 25175 strain with a MIC value of 25 *μ*g/*μ*l and a MBC value of 40 *μ*g/*μ*l, whereas, against* R. dentocariosa*, it has displayed a moderate inhibitory activity, with MIC and MBC values of 20 *μ*g/*μ*l and 140 *μ*g/*μ*l, respectively.

**Conclusions:**

* In vitro *microbiological tests demonstrate that the hydroalcoholic extracts of pomegranate juice and peel are able to contrast the main cariogenic bacteria involved in tooth decay. Although being preliminary data, our results suggest that pomegranate polyphenolic compounds could represent a good adjuvant for the prevention and treatment of dental caries.

## 1. Introduction

Even though the prevalence of dental caries has decreased through the use of preventive systems (fluoride prophylaxis, fluoride toothpastes, control of oral hygiene, and sealants) [1–4], it still remains one of the most common chronic diseases both in health and in systemic diseases affected children [5–7]. The etiology of tooth decay is multifactorial and it is induced by three main factors: host, environment, and bacteria. Today it is known that different bacterial species cause the strongest effect on the prevalence and incidence of dental caries.

*Streptococcus mutans *represents one of the main factors for triggering of dental caries, because it can adhere to tooth surfaces and produce large amounts of acid. The key virulence factors are the water-insoluble glucan synthesized from sucrose and involved in biofilm formation, the acidogenicity, and finally the acid tolerance [8, 9]. Many studies have revealed that* S. mutans *represents about the 20–40% of the cultivable flora in biofilms removed from carious lesion [10].* Rothia *spp., in particular* R. dentocariosa*, are common inhabitants of the oral cavity. Recent reports suggest that these species could be opportunistic pathogens, causing a number of diseases in addition to dental and periodontal pathologies [11].* R. dentocariosa*, originally isolated from carious lesions of human teeth, has been found to cause endocarditis [12], pneumonia [13], and infections of the peritoneum and lung [14].

*S. mutans *and the other microorganisms involved in the pathogenesis of dental caries have been considered very difficult to control, because they have developed tolerance and resistance to many antimicrobial agents routinely used in the clinical practice [15].

The chlorhexidine has been studied for nearly 40 years primarily for its ability to reduce gingivitis. Classified as an antimicrobial agent, it has been proven to inhibit the formation and development of dental plaque biofilm [16]. However, it can cause a change in taste and produce yellow or brown pigments on tooth surfaces. Therefore, the use of chlorhexidine for caries prevention is controversial, especially in children [16].

The resistance of microorganisms against the antibiotics commonly used to treat oral infections, the increasing number of oral pathologies, and the lack of medications without side effects require identifying new effective strategies against oral pathogens. Since ancient times, the bioactive principles of plant origin have been used for treatment of many diseases and microbial infections. In the last decades, the use of plants with preventive and therapeutic effects contributing to health care has increased. Scientists investigated many plant products in order to find their effectiveness in the prevention of dental plaque formation [17–19].

Numerous medicinal plant extracts have been shown to inhibit the formation of dental biofilms by reducing the adhesion of microbial pathogens to the tooth surface or reducing the number of bacteria implicated in the caries pathogenesis [20, 21]. However, only few natural products have found therapeutic applications. The reasons of such limited use are due to different factors as effectiveness, stability, smell, taste, and, not last, cost [22].

Pomegranate (*Punica granatum *L.) is a common fruit of a tree belonging to the family* Punicaceae*. It is native to the region from northern India to Iran and it has been cultivated and naturalized over the entire Mediterranean region since ancient times. The ripe fruit is about five inches wide with deep red, leathery skin, grenade shape with a pointed calyx. The fruit contains many seeds separated by white membranous pericarp. Each seed is surrounded by tart and red juice [23].

Pharmacological properties of pomegranate have a long history, but, in the recent decades, the interest in evaluating therapeutic effects of pomegranate has increased noticeably. Studies show that pomegranate juice has potent antioxidant activity (capability to scavenge free radicals) due to its high polyphenols content, including ellagitannins (hydrolysable tannins) and anthocyanins (condensed tannins). There is a range of phytochemical compounds in pomegranate that have showed antimicrobial activity, but most of the researchers have found that ellagic acid and larger hydrolyzable tannins, such as punicalagin, have the most important activities. In many cases, the mixture of the pomegranate constituents offers the most advantage [24]. This fruit has also been used in traditional medicine for the treatment of dysentery, diarrhea, and respiratory pathologies [25, 26].

Many studies indicate that pomegranate extracts may be employed as natural alternative for the treatment of a wide range of bacterial and viral infections due to their antimicrobial activity. Recent study indicates that both pomegranate aril and peel extracts have an effective antimicrobial activity, as evidenced by the inhibitory effect on the bacterial growth of two important human pathogens, including* Staphylococcus aureus *and* Escherichia coli*, often involved in foodborne illness [27]. In addition, experimental data strongly support the antibacterial activity of pomegranate extracts against oral pathogen such as* S. mutans *[28]. However, little is known about the effect of pomegranate extracts on other pathogens involved in tooth decay such as* R. dentocariosa*, the first bacterium isolated from carious dentin [29].

The aim of present study was to evaluate the antimicrobial activity of hydroalcoholic extracts of pomegranate (*Punica granatum *L.) peel and juice against* S. mutans *ATCC 25175 strain and* R. dentocariosa *clinical isolates.

## 2. Materials and Methods

### 2.1. Preparation of Extracts for Microbiological Assay

Fresh fruits of pomegranate (*P. granatum *L.) were collected from trees located in the countryside of Avellino (Southern Italy) during fruit season. The fruits were handpicked, washed, and peeled, and the arils, without seeds, were hand-crushed and then squeezed in order to obtain the juice. The peel was air dried a few days and then pulverized. The samples were stored at −20°C for further analysis. The juice was defrosted at room temperature. Solution water/ethanol 25 ml 50% (v/v) was added to 5 g of juice. The same procedure was carried out for the peel powder. Each sample was mixed for 30 minutes, and then the extracts were filtered.

The analysis of phenolic compounds of the pomegranate (juice and peel) was performed by reverse phase HPLC (RP) coupled offline mass spectrometry (MS) MALDI-TOF as described in our previous study [27].

For microbiological assays, the ethanolic extracts of juice and peel were dried in Savant in order to calculate the percentage yield of total polyphenols. Each extract was reduced in volume in a rotavapor, transferred into a plastic tube, and finally lyophilized. The hydroalcoholic extracts of pomegranate peel and juice were used, as described in our previous study [27].

### 2.2. Microorganisms and Growth Conditions

The antimicrobial activity of the pomegranate extracts was evaluated against the strain* Streptococcus mutans *Clarke ATCC 25175 (LGC Standards, UK) isolated from carious dentine and* Rothia dentocariosa *clinical isolate Rd1, obtained from samples of dental plaque provided from the Pediatric Dentistry Department of “Federico II” University, Naples, Italy. Permission to take dental plaque samples was acquired according to the local planning authorities. Furthermore, approval for this study was granted by the ethics committee of the “Federico II” University, Naples, Italy (Protocol number 101/14).

The identification of clinical isolates was performed, from UOC of Clinical Microbiology, AOU “Federico II” of Naples, Italy, by mass spectrometry using the Matrix Assisted Laser Desorption/Ionization (MALDI) mass spectrometer (Bruker Daltonics, MALDI Biotyper, Fremont, CA, USA), a high-throughput proteomic technique for identification of a variety of bacterial and fungal species [30, 31], and biochemical phenotyping method in an BD Phoenix™ Automated Microbiology System (Becton Dickinson, BD Franklin Lakes, NJ, USA), according to the manufacturer's instruction.

Bacteria were cultured aerobically in broth and agar media at 37°C. The media used were Brain Heart Infusion (BHI) (Oxoid, S.p.a., Rodano, Milano, Italy), Columbia CNA with 5% Sheep Blood with Colistin and Nalidixic Acid (Oxoid, S.p.a., Rodano, Milano, Italy), and Mueller-Hinton (Simad s.a.s., Naples, Italy). Microbial strains were maintained at 4°C on agar media. The isolates were stored frozen at −80°C in BHI broth supplemented with 10% glycerol (v/v) (Carlo Erba, Reagents, Milan, Italy) until use and the working cultures were activated in the respective broth at 37°C for 15–18 h.

### 2.3. *In Vitro *Antibacterial Activity Assays

The susceptibility of* S. mutans *ATCC 25175 and* R. dentocariosa *Rd1 to different concentrations of* Punica granatum *L. fruit extracts was determined by dilution tube method with 1 × 10^5^ CFU/ml as standard inoculums [32]. The extracts were added in the series of tubes achieving final concentrations of 0, 5, 10, 15, 20, 30, 40, 60, 100, and 140 *μ*g/*μ*l, and tubes were incubated at 37°C for 24 h. As positive control the bacterial strains were tested with ranging concentrations of Ampicillin (Sigma-Aldrich, Milano, Italy) and with extraction buffer as negative control. After incubation, the optical density at *A*_600 nm_ was determined; subsequently an aliquot of each sample was spread into BHI-agar plates in duplicate and then incubated for 24–48 h for the evaluation of viable counts. Minimum inhibitory concentration (MIC) was assigned to lowest concentration of pomegranate extract, which prevents bacterial growth. The minimum bactericidal concentration (MBC) was defined as the minimum extract concentration that killed 99% of bacteria in the initial inoculums.

To verify the effect of pomegranate juice and peel hydroalcoholic extracts on the fitness of* S. mutans *ATCC 25175 and* R. dentocariosa *Rd1, assays of bacterial growth and survival were performed in presence of increasing concentrations of the extracts. To evaluate the fitness of each strain, during the observation period (96 h), serial dilutions were spread on BHI-agar and incubated at 37°C for 24–48 h to evaluate viable counts. All experiments were performed in triplicate, with three independent cultures; the results obtained were analyzed and graphically reported by using “GraphPad Prism 6” software. Results are presented as mean ± SD. The statistical significance was determined by the two-way ANOVA test with a Bonferroni correction (*P* value ≤ 0.05).

## 3. Results

### 3.1. *In Vitro *Antibacterial Activity of Pomegranate Extracts

The antimicrobial activity of pomegranate extracts against* S. mutans *ATCC 25175 cariogenic strain and* R. dentocariosa *Rd1 clinical isolate was evaluated by dilution tube method, according to the CLSI (Clinical and Laboratory Standards Institute) guidelines [33].

Growth of* S. mutans *ATCC 25175 strain and* R. dentocariosa *Rd1 clinical isolate was inhibited with a concentration of pomegranate juice extract equal to 25 *μ*g/*μ*l and 20 *μ*g/*μ*l, respectively.

Pomegranate juice extracts showed a MBC value of 40 *μ*g/*μ*l against* S. mutans *ATCC 25175 and a MBC value of 140 *μ*g/*μ*l against* R. dentocariosa *Rd1 (Table 1).

The pomegranate peel extracts exhibited a MIC value of 10 *μ*g/*μ*l and a MBC value of 15 *μ*g/*μ*l against both microorganisms tested. Both the bacteria tested in this study are sensitive to ampicillin (Table 1).

### 3.2. Effects of Pomegranate Extracts on Bacterial Fitness

**Figure 1:**
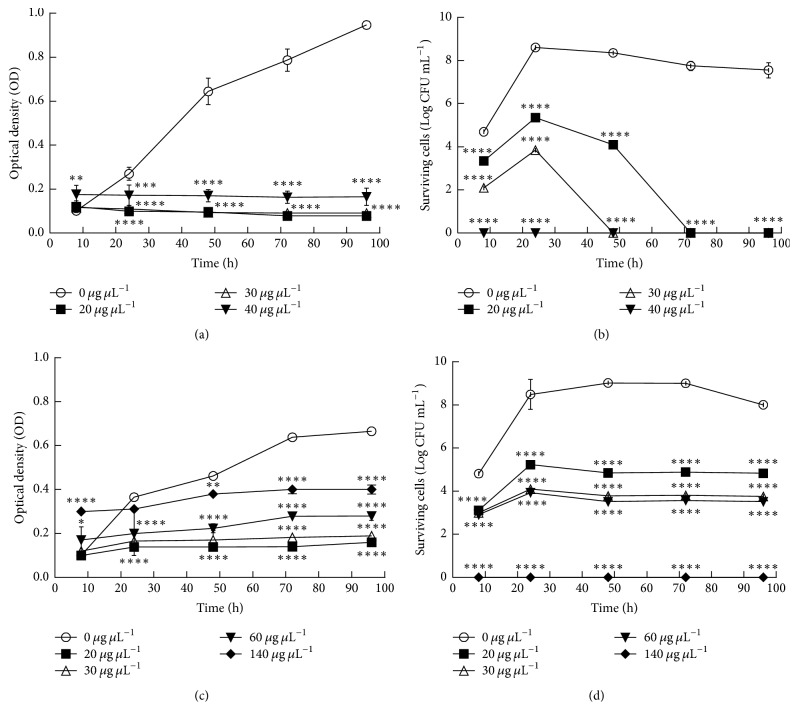
Effect of pomegranate juice extracts on (a) growth of* S. mutans *at different concentration (0, 20, 30, and 40 *μ*g/*μ*l); (b) survival of* S. mutans *at different concentration (0, 20, 30, and 40 *μ*g/*μ*l); (c) growth of* R. dentocariosa *at different concentration (0, 20, 30, 60, and 140 *μ*g/*μ*l); (d) survival of* R. dentocariosa *at different concentration (0, 20, 30, 60, and 140 *μ*g/*μ*l). The experiments were performed in triplicate and statistical significance was examined by the two-way ANOVA test with a Bonferroni correction. Results are indicated as means ± SDs. Asterisks indicate statistical significance (^*∗*, *∗∗*^*P* < 0.05; ^*∗∗∗*^*P* < 0.001; ^*∗∗∗∗*^*P* < 0.0001).

**Figure 2:**
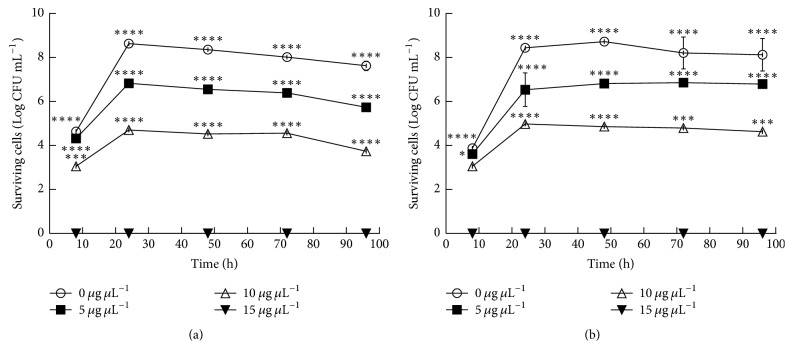
Effect of pomegranate peel extracts on survival of* S. mutans *(a) and* R. dentocariosa *(b) at different concentration (0, 5, 10, and 15 *μ*g/*μ*l). The experiments were performed in triplicate and statistical significance was examined by the two-way ANOVA test with a Bonferroni correction. Results are indicated as means ± SDs. Asterisks indicate statistical significance (^*∗*^*P* < 0.05; ^*∗∗∗*^*P* < 0.001; ^*∗∗∗∗*^*P* < 0.0001).

To verify the effect of pomegranate juice and peel hydroalcoholic extracts on the fitness of* S. mutans *ATCC 25175 cariogenic strain and* R. dentocariosa *Rd1 clinical isolate, the growth and survival were evaluated for 96 h, with increasing concentrations of hydroalcoholic extracts. The pomegranate juice extracts exhibited inhibitory effect on growth and survival of both strains (Figure 1). The growth evaluation was biased by the turbidity of the extracts, as clearly showed by growth curves (Figures 1(a) and 1(c)). However, the evaluation of viable counts had highlighted a strong bactericidal activity of pomegranate juice hydroalcoholic extract with a concentration of 40 *μ*g/*μ*l for* S. mutans *ATCC 25175 and a moderate bactericidal effect against* R. dentocariosa *Rd1 with a concentration of 140 *μ*g/*μ*l (Figures 1(b) and 1(d)). Interestingly, the pomegranate hydroalcoholic peel extract exhibited a strong inhibitory activity against both tested cariogenic strains (Figure 2). The hydroalcoholic peel extracts interfered with the bacterial growth, survival, and fitness in a dose dependent manner and with time-lasting effects, as previously described for other clinical isolates [27]. In addition the bactericidal activity is detectable at a very low concentration equal to 15 *μ*g/*μ*l for both strains. The peel extracts in ethanol were cloudy so it was impossible to test it in the bacterial growth assay.

## 4. Discussion

Results of the present study showed that pomegranate juice and peel extracts were effective against the main cariogenic pathogens such as* S. mutans *ATCC 25175 cariogenic strain and* R. dentocariosa*, Rd1 clinical isolate.

The present research was in line with other studies demonstrating antibacterial agents from plant were effective to prevent and contrast oral and periodontal disease and tooth decay [34–37].

In particular, among plants*, Punica granatum *L., used in traditional medicine, is known for its pharmacological properties that have been evaluated due to antiparasitic, antibacterial, antifungal, antiproliferative, apoptotic, and anticancer effects [38].

Literature data reported that extracts of* Punica granatum *L. peel in different concentrations were effective against different bacterial species such as* S. aureus*,* E. coli*,* Salmonella enterica*,* Shigella sonnei*,* Enterococcus faecalis*, and* Bacillus subtilis *[27, 39]. The amount of total polyphenols varied depending on the parts of the fruit, in particular being higher in the peel than in the juice extracts [27]. According to these findings, our data highlighted that the pomegranate peel extract strongly inhibits the growth and the survival of both cariogenic strains with MIC value of 10 *μ*g/*μ*l and MBC value of 15 *μ*g/*μ*l.

The MIC values of pomegranate extracts determined in different studies significantly vary. For example, the MIC against* S. aureus *isolates are reported to range from 0.62 to >250 *μ*g/*μ*l [27, 40–42]. This variability is not surprising, considering that it is typically recorded even with conventional antimicrobials against all clinical isolates [43], though generally within a more restricted range. Therefore, our results are in agreement with previous studies that established that pomegranate extracts could reduce the viable count of* S. mutans *to a degree equivalent to that of 1.2% CHX mouthwash [44, 45].

In 2006, also Vasconcelos et al. investigated the antimicrobial effect of* Punica granatum *L. (pomegranate) using a phytotherapeutic gel, pomegranate based, and miconazole (Daktarin® oral gel) against three standard streptococci strains (*mutans *ATCC 25175,* sanguis *ATCC 10577, and* mitis *ATCC 9811) and demonstrated the greater efficiency of pomegranate gel in inhibiting microbial adherence than miconazole [46].

While the antibacterial activity of the pomegranate peel has been the subject of numerous researches, few studies have investigated the antibacterial activity of pomegranate juice against oral pathogens, such as* S. mutans *and* R. dentocariosa*. Kote and Nagesh in 2011 conducted a clinical trial that showed the ability of pomegranate juice to reduce the microorganisms of dental plaque (streptococci and lactobacilli) [47].

This* in vitro *study demonstrates that* Punica granatum *L. peel and juice extracts are efficacious against cariogenic bacteria, supporting the hypothesis that pomegranate polyphenols could exert an anticaries effect via an antimicrobial mode-of-action.

In addition, our findings demonstrate for the first time the inhibitory effect of hydroalcoholic pomegranate extracts on* R. dentocariosa*, isolated from dental plaque, a bacterium considered as one of the main etiological agents of several oral diseases, including tooth decay [5, 48, 49].

## 5. Conclusions

*In vitro *microbiological assays demonstrated that pomegranate (*Punica granatum *L.) hydro-alcoholic peel and juice extracts are able to counteract cariogenic bacteria of dental plaque. In fact, the extracts showed inhibitory effect on the growth and survival of* S. mutans *ATCC 25175 and* R. dentocariosa *Rd1 isolate, considered among the most important etiological agents of tooth decay. The strongly bactericidal power of the pomegranate fruit extracts against oral cariogenic bacteria suggests further deep investigation.

Much of the evidence for pomegranates antibacterial and antiviral activities against foodborne pathogens and other organisms responsible of infectious disease comes from* in vitro *cell based assays, necessitating further confirmation of* in vivo *efficacy through human clinical trials. Therefore, more controlled trials using different concentrations of pomegranate fruit extracts are necessary to verify its action upon supragingival microflora* in vivo*. Within the limits of the present study, it may be concluded that the pomegranate fruit extracts are effective against dental plaque microorganisms. Further research is needed to identify the real benefits of pomegranate as a therapeutic and preventive agent for dental plaque microorganisms and to identify the specific active constituents in pomegranates that could be useful as anticaries/antiplaque agents and the safer dose that can be used in humans.

## Figures and Tables

**Table 1:** Antibacterial activity of pomegranate fruit extracts against *Streptococcus mutans *and *Rothia dentocariosa.*

Cariogenic bacteria	Pomegranate juice extracts (*μ*g/*µ*l)		Pomegranate peel extracts (*μ*g/*µ*l)		Ampicillin (*μ*g/*µ*l)	
	MIC^a^	MBC^b^	MIC	MBC	MIC	MBC
*S. mutans *ATCC 25175	25	40	10	15	0.01	0.02
*R. dentocariosa* Rd1	20	140	10	15	0.04	1.2

^a^MIC: minimum inhibitory concentration. ^b^MBC: minimum bactericidal concentration.
